# Combined Radio- and Chemotherapy for Non-Small Cell Lung Cancer: Systematic Review of Landmark Studies Based on Acquired Citations

**DOI:** 10.3389/fonc.2013.00176

**Published:** 2013-07-09

**Authors:** Carsten Nieder, Adam Pawinski, Nicolaus H. Andratschke

**Affiliations:** ^1^Department of Oncology and Palliative Medicine, Nordland Hospital, Bodø, Norway; ^2^Institute of Clinical Medicine, Faculty of Health Sciences, University of Tromsø, Tromsø, Norway; ^3^Department of Radiation Oncology, University Hospital Rostock, Rostock, Germany

**Keywords:** chemoradiation, chemotherapy, citation, non-small cell lung cancer, radiotherapy, research evaluation

## Abstract

The important role of combined chemoradiation for several groups of patients with non-small cell lung cancer (NSCLC) is reflected by the large number of scientific articles published during the last 30 years. Different measures of impact and clinical relevance of published research are available, each with its own pros and cons. For this review, article citation rate was chosen. Highly cited articles were identified through systematic search of the citation database Scopus. Among the 100 most often cited articles, meta-analyses (*n* = 5) achieved a median of 203 citations, guidelines (*n* = 7) 97, phase III trials (*n* = 29) 168, phase II trials (*n* = 21) 135, phase I trials (*n* = 7) 88, and others combined 115.5 (*p* = 0.001). Numerous national and international cooperative groups and several single institutions were actively involved in performing often cited, high-impact trials, reflecting the fact that NSCLC is a world-wide challenge that requires research collaboration. Platinum-containing combinations have evolved into a standard of care, typically administered concurrently. The issue of radiotherapy fractionation and total dose has also been studied extensively, yet with less conclusive results. Differences in target volume definition have been addressed. However, it was not possible to test all theoretically possible combinations of radiotherapy regimens, drugs, and drug doses (lower radiosensitizing doses compared to higher systemically active doses). That is why current guidelines offer physicians a choice of different, presumably equivalent treatment alternatives. This review identifies open questions and strategies for further research.

## Background

Combined radio- and chemotherapy has since the 1980s evolved into a standard of care for a considerable proportion of patients with stage III non-small cell lung cancer (NSCLC) (1–3). Importantly, stage III describes a heterogeneous population with disease presentation ranging from apparently resectable tumors with occult microscopic nodal metastases to unresectable, large volume nodal disease. Even discriminating between stage IIIA and B does not fully resolve this problem. One of the most controversial issues in patients potentially accessible for surgery is the role of neoadjuvant therapy (4). As recently summarized, neoadjuvant therapy followed by surgery is neither clearly better nor clearly worse than definitive chemoradiation (5). Most of the arguments made regarding patient selection for neoadjuvant therapy and surgical resection provide evidence for better prognosis but not for a beneficial impact of this treatment strategy. We will not discuss in greater detail the emerging role of image-guided stereotactic radiotherapy as boost or salvage treatment (6, 7) because most data on this modality were derived from studies of stage I NSCLC, where ongoing randomized trials compare surgery to stereotactic radiotherapy (8–11). Both individual institutions and cooperative groups successfully completed an impressive series of clinical trials for advanced NSCLC, many of whom resulted in practice-changing insights. For several reasons including but not limited to tenure track or likelihood of future funding, researchers attempt to publish their results in a way that ensures high visibility and allows for broad adoption of the progress achieved. Landmark studies often appear in prestigious high-impact journals, and are likely to be cited in editorials, reviews, guidelines, etc., (12). Number of acquired citations might be a measure that can be used when performing systematic reviews because it eliminates subjective preferences when selecting influential research to be included (13). For the present review, we relied on citation-based selection of studies, and secondary we hypothesized that randomized clinical trials and meta-analyses acquired more citations than other types of research.

## Materials and Methods

A systematic search of the citation database Scopus (Elsevier B.V., www.scopus.com) by use of the term “radiotherapy and lung cancer” was performed on 16th March 2013. Articles were selected irrespective of language, year of publication, and article type (review, guideline, clinical study, experimental study, etc.). In order to determine whether or not a given article reported on NSCLC and combined radio- and chemotherapy we accessed its abstract if the title was not sufficiently informative. Then, all articles dealing with the subject of this review were ranked by number of citations (field “times cited” in the Scopus citation database) in order to create a list (top 100) of articles with the highest number of citations. The top 100 articles were reviewed for contents incl. study type (phase I, II, III, retrospective, etc.) and outcomes. Moreover, the following parameters were evaluated: journal in which an article was published, number of authors, and type of research. A complete list of top 100 articles can be requested from the corresponding author.

## Results

**Figure 1:**
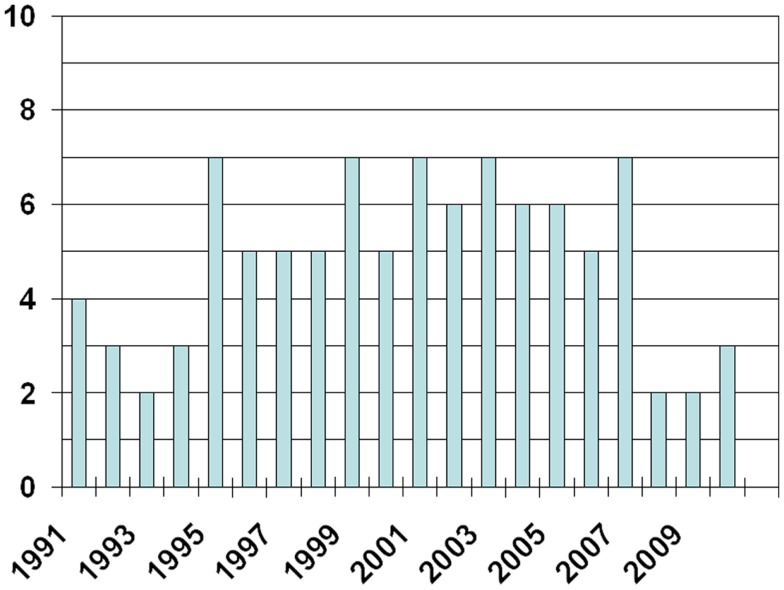
**Number of articles published per year**.

The 100 most often cited articles were published between 1984 and 2010 (Figure 1, only three articles were published before 1991). They achieved a median number of 124 citations (range 66–2733). Articles published before 2000 achieved a median of 137 citations (range 66–2733). Those published in the time period 2000–2010 achieved a median of 100 citations (range 66–985). In order to cover the most recent results and trends, we also extracted the top 10 publications from the year 2011 (14–23). They achieved a median of 14.5 citations (range 12–34). References (24–48) represent the 25 most cited articles overall. With regard to the top 100 publications, 3 were written by more than 20 authors. Eight had 15–20 authors, 36 had 10–14 authors, the majority (41) had 5–9 authors, and 12 had less than 5 authors (median number 9, range 1–35). Twenty-nine articles reported on phase III clinical trials (phase II: 21, phase I: 7), five were meta-analyses, seven guidelines, and two literature reviews. The remaining were retrospective and preclinical studies. Meta-analyses achieved a median of 203 citations, guidelines 97, phase III trials 168, phase II trials 135, phase I trials 88, and others combined 115.5 (*p* = 0.001, chi square test). Most articles were published in the Journal of Clinical Oncology (*n* = 43), the International Journal of Radiation Oncology Biology and Physics (*n* = 15), the Journal of the National Cancer Institute and the Journal of Thoracic and Cardiovascular Surgery (*n* = 5 each), Clinical Cancer Research and Annals of Thoracic Surgery (*n* = 4 each), as well as Chest (*n* = 3). Five publications on major randomized clinical studies emanated from US or US and Canadian intergroup trials, six from SWOG trials, five from CALGB trials, and four from RTOG trials. Between one and three publications were related to trials performed by ECOG, EORTC, Hoosier Oncology Group, California Cancer Consortium, and different other groups from Australia, China, Europe, and Japan. Cooperative group studies accumulated a median of 154 citations compared to 107 citations for studies not performed by cooperative groups (*p* = 0.02).

## Discussion

The objective of this review was to identify influential, highly cited scientific publications and thereby research mainstreams related to chemoradiation treatment for NSCLC. After arbitrary decisions about which database to search and which keywords to use, we performed a systematic literature search. Citation rate of published articles was evaluated. Articles with high numbers of citations are likely those that impressed other clinicians/scientists and had profound influence on clinical practice or future developments in the field. It should be noticed that searches in different databases will result in more or less variable citation counts and that the present results therefore provide only a snapshot. Our results are consistent with the assumption that citation rate is gradually increasing for several years after publication. However, the purpose of this overview was not to explore dynamics of citation count. Meta-analyses and phase III trials were the publications accumulating the highest numbers of citations, a finding already described for other types of cancer (13). However, we focused on top 100 articles rather than all meta-analyses and clinical trials on chemoradiation. All major international cooperative groups were actively involved in often cited, high-impact trials, reflecting the fact that NSCLC is a world-wide challenge requiring research collaboration. Cooperative group trials achieved a significantly higher median number of citations compared to other trials. Given the complexity of large clinical trials, which often include companion biomarker studies, and require rapid patient accrual, cooperative groups with their dedicated infrastructure might have advantages in conducting these important studies with potentially practice-changing implications. Typically, convincing phase III data are required before new therapeutic approaches are adopted in the oncology community. However, exceptions from this rule are possible, for example the rapid and widespread use of stereotactic radiotherapy for stage I NSCLC or approval of crizotinib in patients with rearrangements of anaplastic lymphoma kinase (ALK) gene.

The interest in combined modality treatment is old and caused by the fact that neither treatment modality by itself provided satisfactory clinical outcomes. Studies performed in the 1980s often examined non-concomitant approaches [induction chemotherapy followed by radiation (28) or pre and post irradiation chemotherapy (49)]. However, initial concomitant approaches also date back to this time period (27, 50). Platinum-containing combinations have evolved into a standard of care (26, 27, 35), typically administered concurrently (14, 24, 51). The issue of radiotherapy fractionation has also been studied extensively (conventional daily 1.8–2 Gy fractions to approximately 60–66 Gy, hyperfractionation, acceleration by more than one daily fraction, acceleration by hypofractionation) (23, 34, 39). So far, conventional fractionation continues to be equivalent to altered fractionation. Overall treatment time increases when attempting dose escalation with conventional fractionation, and this factor might limit the efficacy of numerically more intense radiotherapy regimens (18). One possibility to reduce overall treatment time is the use of hypofractionation, either during the whole course of chemoradiation or for example by use of stereotactic boost radiotherapy (52, 53), thereby administering a high biologically effective dose within a standard time frame. The same problem of accelerated repopulation of cancer cells during prolonged treatment could explain the lack of benefit from sequential chemo- and radiotherapy as compared to concurrent administration (51). Stereotactic radiotherapy is now also being studied for patients with locally recurrent disease (54). Differences in target volume definition have been addressed [avoiding elective lymph node regions (55), integrating positron emission tomography (PET) information (22)]. However, it was not possible to test all theoretically possible combinations of radiotherapy regimens, drugs, and drug doses (lower radiosensitizing doses compared to higher systemically active doses). That is why current guidelines offer physicians a choice of different, presumably equivalent treatment alternatives (56), which we will not discuss and repeat in detail.

Introduction of more sophisticated staging incl. PET, refinement of TNM categories, and increasing frequency of non-squamous cell carcinoma histologies make it difficult to compare contemporary and historic patient groups. The prognostic and predictive impact of primary and total tumor volume, number of involved lymph nodes, and pattern of lymphatic spread incl. number of involved stations needs to be determined in a more rigorous fashion (57). Moreover, NSCLC is a biologically heterogeneous group of diseases. Even if molecular categories continue to evolve, therapeutic implications are already evident (use of pemetrexed or epidermal growth factor receptor inhibitors, and more targeted agents are currently being tested). As seen with bevacizumab (58), toxicity of full dose platinum-based chemoradiation plus additional agents might be prohibitive, depending on pathways targeted by new agents. The picture gets even more complicated when one adds host heterogeneity (age, comorbidity, nutrition status). Clearly, not all patients are suitable for the most efficacious concurrent regimens (59). In such cases, sequential treatment still might be considered, as is the case when initial tumor volume would necessitate prohibitively high normal lung dose.

At present, many open questions remain. Does addition of surgery to chemoradiation [an intensely studied paradigm (29, 36, 41, 43, 46)] provide better outcomes, and if so in patients with primarily resectable stage III disease and those with more advanced disease but favorable response to induction? Or is this approach relevant only for superior sulcus tumors (60)? Is radiation dose escalation still promising or a futile approach that only increases toxicity (18)? Esophagitis and pneumonitis are well known dose-limiting toxicities (61–64). Can proton therapy overcome limitations of other techniques (19, 21)? Will pharmacological toxicity mitigation strategies ever make it into routine clinical practice (65)? Will innovative concepts of consolidation chemotherapy after chemoradiation perform better than previous attempts (37, 66, 67)? Is there a role for PET not only prior to treatment but also during chemoradiation, predicting efficacy, or allowing for treatment plan adaptation (17)? Can we identify those elderly patients who will tolerate intense combined modality treatment (68)? Both local failure resulting from surviving tumor stem cells and development of distant metastases contribute to the modest long-term survival after chemoradiation (69). Some of the studies reviewed here attempted to improve only one, others both sources of failure. Development of brain metastases is a threat to many patients with initial stage III disease. So far, prophylactic cranial radiotherapy has not improved overall survival (70, 71). Despite these challenges, progress has been achieved and much has been learned. Technological, molecular, and pharmacological progress provides a basis for new generations of clinical trials. Continued support by patients, health care providers, payors, and sponsors is necessary to pursue the bumpy yet successful way toward survival improvement.

## Conclusion

Citation count might aid individuals who try to identify important studies to achieve this goal. Progress in chemoradiation development was largely driven by cooperative groups and some dedicated single institutions, which pursued their concepts through different stages of clinical trials, often culminating in successful phase III trials. The latter as well as meta-analyses are likely to change clinical practice and achieve high citation counts.

## Conflict of Interest Statement

The authors declare that the research was conducted in the absence of any commercial or financial relationships that could be construed as a potential conflict of interest.
